# Flutter Atrial na Síndrome do PRKAG2: Características Clínicas e Eletrofisiológicas

**DOI:** 10.36660/abc.20210792

**Published:** 2022-09-06

**Authors:** Eduardo Faria Soares de Magalhães, Luiz Pereira de Magalhães, Jussara de Oliveira Pinheiro, Alex Teixeira Guabiru, Roque Aras

**Affiliations:** 1 Universidade Federal da Bahia Faculdade de Medicina de Bahia Salvador BA Brasil Universidade Federal da Bahia – Faculdade de Medicina de Bahia , Salvador , BA – Brasil; 2 Hospital Universitário Professor Edgard Santos Salvador BA Brasil Hospital Universitário Professor Edgard Santos , Salvador , BA – Brasil

**Keywords:** Arritmias Cardíacas, Flutter Atrial, Hipertrofia Ventricular Esquerda, Cardiomiopatia Hipertrófica, Bloqueio Atrioventricular, Doença de Depósito de Glicogênio

## Abstract

**Fundamento:**

A síndrome do PRKAG2 é uma rara doença genética autossômico dominante, fenocópia da miocardiopatia hipertrófica, caracterizada pelo acúmulo intracelular de glicogênio. Manifestações clínicas incluem pré-excitação ventricular, hipertrofia ventricular, distúrbio de condução cardíaca e arritmias atriais.

**Objetivo:**

Comparar características clínicas e eletrofisiológicas observadas em pacientes com flutter atrial, com e sem síndrome do PRKAG2.

**Métodos:**

Estudo observacional, comparativo de pacientes com flutter atrial: grupo A, cinco pacientes de família com síndrome do PRKAG2; e grupo B, 25 pacientes sem fenótipo da síndrome. O nível de significância foi de 5%.

**Resultados:**

Todos os pacientes do grupo A apresentaram pré-excitação ventricular e bloqueio de ramo direito; quatro tinham marca-passo (80%). Pacientes do grupo A tinham menor idade (39±5,4 vs. 58,6±17,6 anos, p=0,021), e maior espessura de septo interventricular (mediana=18 vs. 10 mm; p<0,001) e parede posterior (mediana=14 vs. 10 mm; p=0,001). Quatro do grupo A foram submetidos a estudo eletrofisiológico, sendo observada via acessória fascículo-ventricular; em três foi realizada ablação do flutter atrial. Todos os do grupo B foram submetidos à ablação do flutter atrial, sem evidência de via acessória. Observado maior prevalência no grupo B de hipertensão arterial, diabetes mellitus, doença coronariana e apneia do sono, sem diferença estatisticamente significante.

**Conclusão:**

Portadores da síndrome do PRKAG2 apresentaram flutter atrial em idade mais precoce, e menos comorbidades, quando comparados a pacientes com flutter atrial sem fenótipo da mutação. Importante suspeitar de miocardiopatia geneticamente determinada, como síndrome do PRKAG2, em jovens com flutter atrial, especialmente na presença de pré-excitação ventricular e hipertrofia ventricular familiar.

## Introdução

A síndrome do PRKAG2 é uma doença genética rara, de herança autossômica dominante, causada por mutações no gene que codifica a subunidade γ _2_ da proteína quinase AMP-ativada (AMPK). ^1 , 2^ O principal achado histopatológico no coração de pacientes acometidos é o depósito intracelular de glicogênio no miocárdio, podendo desencadear alterações eletrofisiológicas e estruturais cardíacas que mimetizam a síndrome de Wolff-Parkinson-White (WPW) e miocardiopatia hipertrófica. ^3^ Estudos indicam que a prevalência da síndrome do PRKAG2 seja de 0,23 a 1,4% dos pacientes com suspeita de miocardiopatia hipertrófica. ^3 , 4^ Possivelmente a incidência da síndrome do PRKAG2 permanece subestimada na prática clínica, pois muitos casos podem ser equivocadamente diagnosticados como miocardiopatia hipertrófica sarcomérica.

A manifestação fenotípica da síndrome do PRKAG2 tem grande variabilidade, consistindo em pré-excitação ventricular, hipertrofia ventricular esquerda, distúrbios do sistema de condução, e taquiarritmias atriais. ^5^ A identificação precoce da síndrome do PRKAG2 é de particular interesse, pois está relacionada a alto risco de evolução para bloqueio atrioventricular total com necessidade de implante de marca-passo, e morte súbita. ^6^

Em relação às taquiarritmias atriais, estudos prévios demonstraram que o flutter atrial é menos comum que a fibrilação atrial na população geral, acometendo mais homens. ^7^ Do ponto de vista eletrocardiográfico, é tipicamente caracterizado pela presença de ondas F (aspecto serrilhado) nas derivações inferiores e frequência atrial aproximada de 300 bpm. ^8^ O mecanismo eletrofisiológico do flutter atrial envolve a macrorreentrada nos átrios, por mecanismo de anisotropia, utilizando áreas de condução lenta, anatômicas ou funcionais. A incidência aumenta com a idade, sendo inferior a 5 casos por 100.000 habitantes entre as pessoas com menos de 50 anos, mas alcançando quase 600 casos por 100.000 habitantes entre as pessoas com mais de 80 anos, com maior risco de fenômenos embólicos. ^9^ Embora a síndrome de WPW e a miocardiopatia hipertrófica estejam relacionadas ao aumento da prevalência de fibrilação atrial, a associação de flutter atrial e pre-excitação ventricular é um fenômeno raro. ^10 , 11^ Na síndrome do PRKAG2, há relatos na literatura de surgimento de flutter atrial em pacientes com pré-excitação ventricular, ^12^ porém são escassos os dados sobre comportamento clínico e eletrofisiológico do flutter atrial na doença. Apesar do flutter atrial poder ser a primeira manifestação da síndrome do PRKAG2, pode ocorrer diagnóstico tardio da síndrome, especialmente quando não há evidente hipertrofia ventricular. ^13^

Este trabalho se propôs comparar as características clínicas, eletrocardiográficas e eletrofisiológicas observadas em pacientes com flutter atrial, com e sem a síndrome do PRKAG2. Para tanto, foram analisados membros de uma mesma família, portadores de flutter atrial e previamente genotipados com a mutação Arg302Gln do gene *PRKAG2* , e comparados com grupo controle de pacientes portadores de flutter atrial sem o fenótipo da síndrome do PRKAG2.

## Métodos

### Participantes do estudo

Trata-se de estudo observacional, retrospectivo, comparativo entre pacientes com flutter atrial, selecionados através de amostragem por conveniência. Os critérios de inclusão foram: (1) pacientes portadores de mutação Arg302Gln do gene *PRKAG2* que evoluíram com flutter atrial; (2) pacientes com registro de flutter atrial isolado do nosso serviço de arritmia nos últimos 5 anos, que foram submetidos à ablação por cateter. Os pacientes foram divididos em dois grupos. O grupo A foi composto de 5 pacientes de uma mesma família acompanhada em nosso serviço, composta de 16 membros portadores de síndrome do PRKAG2, previamente genotipados, ^14^ que apresentaram flutter atrial, com tempo de seguimento médio de 15,1 ± 2,9 anos. O Grupo B incluiu 25 pacientes portadores de flutter atrial típico, sem o fenótipo da síndrome do PRKAG2, consecutivamente submetidos a ablação por cateter, no período de 2015 a 2020.

Foram obtidos dados de exame físico, exames laboratoriais, eletrocardiograma, ecocardiograma com doppler color, e estudo eletrofisiológico. Quanto às características eletrocardiográficas, o intervalo PR curto no eletrocardiograma foi determinado quando era menor que 120 ms. O padrão de pré-excitação ventricular foi definido pela associação de intervalo PR curto com aumento da duração do QRS (> 110 ms) ou empastamento inicial do QRS, mimetizando onda delta. O flutter atrial típico ao ECG foi definido pela presença de ondas F negativas (aspecto serrilhado) em DII, DIII e aVF e positivas em V1. O ECG foi realizado em 12 derivações, na velocidade de 25mm/s, ganho de 10mm:1mV e filtro de 0,05Hz a 15Hz. ^8^

O diagnóstico de hipertrofia ventricular esquerda no ecocardiograma foi estabelecido diante de espessura do septo interventricular ou da parede posterior do ventrículo esquerdo ≥ 13 mm, sem outra causa aparente. ^15^ O estudo eletrofisiológico e ablação por cateter foram realizados através de protocolo de estimulação atrial e ventricular, com uso de cateteres diagnósticos multipolares e cateter de ablação irrigado ou de ponta de 8 mm. A ablação do istmo cavo-tricuspídeo foi realizada bloqueio bidirecional, e demonstração de duplo potencial atrial (> 100 ms). ^16^

### Análise estatística

Após a coleta das informações, os dados foram armazenados em planilha Excel e submetidos à análise estatística, *realizada com software R (R core Team, Vienna, Áustria) para Windows, de acesso gratuito. Durante a análise estatística descritiva,* variáveis categóricas foram expressas em frequência absoluta e porcentagem (%). Variáveis contínuas foram expressas através de média ± desvio-padrão, ou mediana (intervalo interquartil) nos casos de distribuição não normal. Avaliou-se a normalidade dos dados através do teste de Shapiro-Wilk. O teste exato de Fisher foi utilizado para comparação de variáveis categóricas. Variáveis contínuas foram comparadas por meio do teste t de Student para amostras independentes ou pelo teste U de Mann-Whitney, conforme apropriado. *Um valor de p < 0,05 foi considerado significante.*

### Questões éticas

O estudo foi aprovado pelo Comitê de Ética em Pesquisa sob o número 3.044.277, e termo de consentimento livre e esclarecido foi obtido dos participantes.

## Resultados

### Características clínicas

**Figura 1:**
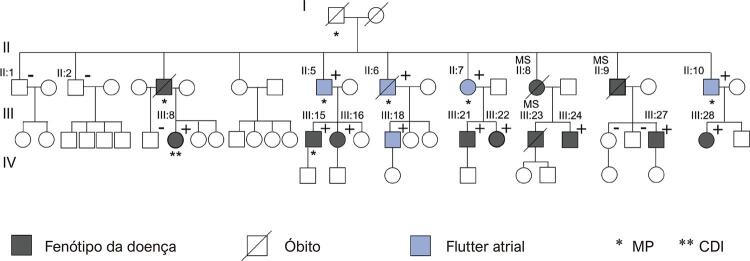
Heredograma da família portadora da síndrome do PRKAG2, com identificação dos 5 pacientes com flutter atrial. MP: marca-passo; CDI: cardioversor desfibrilador implantável. Indivíduos com genotipagem para mutação do gene PRKAG2 identificados como portadores (+) ou não portadores (-). Uma paciente foi submetida a implante de CDI, devido a diagnóstico equivocado de miocardiopatia hipertrófica sarcomérica.

**Figura 2:**
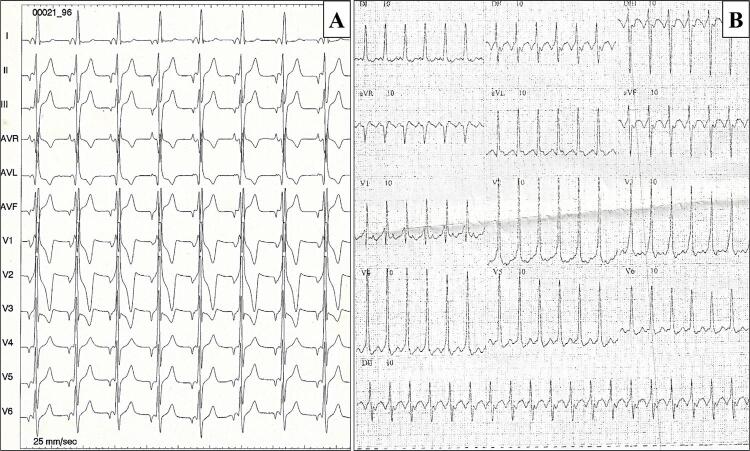
A) Eletrocardiograma inicial do paciente III:15, evidenciando ritmo atrial ectópico, e o aspecto de pré-excitação ventricular, com intervalo PR curto seguido por pseudo-onda delta, e complexo QRS com morfologia de BRD. B) Eletrocardiograma do paciente II:10 com padrão típico da síndrome de PRKAG2, sendo evidenciado flutter atrial com condução 2:1.

As características clínicas, eletrocardiográficas e ecocardiográficas dos pacientes do grupo A, portadores de síndrome do PRKAG2, estão dispostas nas tabelas 1 . A análise do heredograma da família, na Figura 1 , evidencia o padrão de herança autossômico dominante da doença, com relato de três mortes súbitas inexplicadas na família, em indivíduos com idade mediana de 38 anos. Em todos os 5 pacientes incluídos neste estudo, o flutter atrial foi identificado como a primeira manifestação clínica da doença, com idade média ao diagnóstico de 39±5,4 anos. No eletrocardiograma, todos os pacientes do grupo A apresentaram padrão eletrocardiográfico compatível com pré-excitação ventricular, associado a bloqueio de ramo direito (BRD) em quatro (80%). A Figura 2A mostra eletrocardiograma típico de paciente com mutação do *PRKAG2* em ritmo sinusal, e a figura 2B demonstra traçado em ritmo de flutter atrial. Evolução para disfunção do nó sinusal ou bloqueio atrioventricular levaram ao implante de marca-passo em 4 (80%), com idade média ao implante de 44 ± 6 anos.

**Tabela 1:** Características clínicas dos pacientes do grupo A

Paciente	Sexo	Idade	Idade no diagnóstico	Sintomas	Taquiarritmia	MP	HVE
II:5	M	56	30	Palpitação	FLA, FA	+	+
II:6	M	60	42	Síncope, palpitação	FLA, FA	+	+
II:7	F	58	40	Síncope	FLA	+	+
II:10	M	53	44	Pré-síncope, palpitação	FLA	+	+
III:18	M	43	39	Palpitação	FLA	-	-

*M: masculino; F: feminino; MP: marca-passo; HVE: hipertrofia ventricular esquerda; FLA: flutter atrial; FA: fibrilação atrial.*

O grupo B consistiu em 25 pacientes portadores de flutter atrial típico, sendo 19 (76%) homens, seis (24%) assintomáticos, com idade média ao diagnóstico de 58,6±17,6 anos, sendo um (4%) portador de marca-passo por disfunção do nó sinusal. Foi documentado comunicação interatrial em 3 (12%), e cardiopatia induzida por flutter atrial em 3 (12%). Apenas 2 (8%) pacientes do Grupo B apresentaram bloqueio de ramo direito, e nenhum tinha pré-excitação ventricular.

### Aspectos eletrofisiológicos

**Figura 3:**
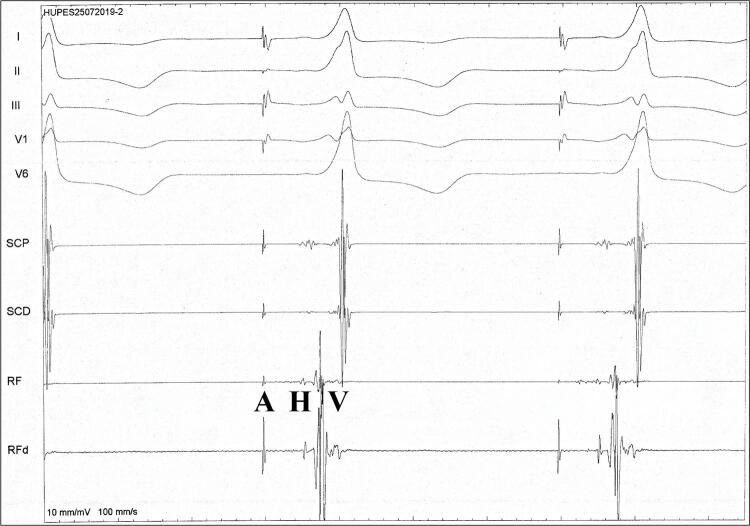
Traçado do estudo eletrofisiológico da paciente II:10. Eletrocardiograma de 5 derivações e intervalos *básicos durante estimulação atrial, demonstrando HV = 33 ms. AV: átrio-ventricular; A: eletrograma atrial; H: eletrograma do His; V: eletrograma ventricular.*

Quatro pacientes do grupo A foram submetidos a estudo eletrofisiológico, sendo três do sexo masculino. Foram registrados intervalos AH e HV curtos, com HV fixo (mediana=30 ms), durante ritmo básico e estimulação atrial rápida ( figura 3 ). O ponto de Wenckebach foi obtido em quatro pacientes, com média de 302,5 ± 31 ms. O teste com adenosina foi realizado, com registro de bloqueio AV anterógrado e retrógrado. Foi observada condução retrógrada ventrículo-atrial nodal decremental durante estimulação ventricular em todos. Os achados foram compatíveis com a presença de via acessória fascículo-ventricular. Durante o procedimento, três pacientes foram submetidos à ablação do flutter atrial, sendo demonstrado circuito arritmogênico dependente do istmo cavo-tricuspídeo. Foi obtido sucesso em 100%, sem recorrência após 18 meses. Todos os pacientes do Grupo B foram submetidos à ablação com cateter do flutter atrial típico istmo cavo-tricuspídeo dependente. Não foi evidenciado presença de via acessória.

### Aspectos comparativos dos grupos A e B

Foram comparadas as características clínicas, eletrocardiográficas, ecocardiográficas dos grupos A e B, dispostas na tabela 2 . A média de idade ao diagnóstico de flutter atrial do grupo A foi significativamente menor que o grupo B (39 ± 5,4 vs. 58,6 ± 17,6 anos; p = 0,021). Dentre os sintomas, foi observado maior prevalência de síncope/pré-síncope no grupo A (p = 0,004). Fatores de risco estabelecidos para desenvolvimento de flutter atrial, tais como hipertensão arterial sistêmica, diabetes mellitus, apneia do sono, obesidade e doença arterial coronariana, foram mais prevalentes no grupo B, porém sem significância estatística. Não foi observada diferença estatisticamente significante entre os grupos em relação à função renal.

**Tabela 2:** Resultado comparativo das características clínicas, eletro e ecocardiográficas dos dois grupos

Características	Grupo A n = 5	Grupo B n = 25	Valor de p
Idade (anos)	54 ± 6,7	60 ± 17,2	0,422
Idade ao diagnóstico (anos)	39,0 ± 5,4	58,6 ± 17,6	0,021
Sexo masculino	4 (80)	19 (76)	0,999
Síncope/Pré-síncope	3 (60)	1 (4)	0,009
HAS	3 (60)	16 (64)	0,999
DM	0 (0)	3 (12)	0,999
Apneia do sono	0 (0)	1 (4)	0,999
Obesidade, IMC > 30 kg/m ^2^	0 (0)	8 (32)	0,287
DAC	0 (0)	4 (16)	0,999
Clearance de creatinina (mL/min/1,73m ^2^ )	77,2 (60,7-81,5)	84,4 (66,0-102,8)	0,275
AE (mm)	42 (38-47)	40 (38-42)	0,435
Septo (mm)	18 (14-26)	10 (9-11)	<0,001
PP (mm)	14 (11-15)	10 (9-11)	0,001
DDVE (mm)	46 (44-50)	50 (47-55)	0,124
FE (%)	71 (60-76)	66 (59-69)	0,223
HVE	4 (80)	1 (4)	0,009
FC (bpm)	52 (44-57)	62 (56-75)	0,007
Intervalo PR (ms)	100 (100-110)	160 (140-188)	<0,001
QRS (ms)	120 (100-140)	90 (90-93)	0,001
BRD	3 (60)	2 (8)	0,022
BAVT	3 (60)	0 (0)	0,002
Marca-passo	4 (80)	1 (4)	0,001

*Variáveis contínuas foram expressas como média ± DP, ou mediana (intervalo interquartil). Variáveis categóricas foram expressas como n (%). HAS: hipertensão arterial sistêmica; DM: Diabetes Mellitus; DAC: doença arterial coronariana; IMC: índice de massa corpórea; FC: frequência cardíaca; BRD: bloqueio de ramo direito; BAVT: bloqueio atrioventricular total; AE: átrio esquerdo; S: septo; PP: parede posterior; DDVE: diâmetro diastólico do ventrículo esquerdo; FE: fração de ejeção; HVE: hipertrofia ventricular esquerda.

Ao eletrocardiograma basal em ritmo sinusal, foi observado que o grupo A apresentava menor frequência cardíaca, intervalo PR mais curto e maior prevalência de BRD. Em relação às características ecocardiográficas, foi observada hipertrofia ventricular esquerda em 80% dos pacientes do grupo A, e em apenas 6% do grupo B (p=0.001). Não houve diferença estatisticamente significante em relação à fração de ejeção de VE e tamanho de átrio esquerdo.

Além disso, foi observado bloqueio atrioventricular total apenas nos pacientes do grupo A (80% vs. 0%, p < 0,001), assim como mais frequentemente necessitaram de implante de MP em relação ao grupo B (80% vs. 8%, p = 0,002).

## Discussão

A síndrome do PRKAG2 é uma rara fenocópia da miocardiopatia hipertrófica, simulando também a síndrome de WPW. ^17^ No entanto, a distinção é crucial, pois a história natural, prognóstico e, em alguns casos, as estratégias de tratamento são marcadamente diferentes. ^18^ Manifestações clínicas da síndrome incluem taquiarritmias atriais como flutter atrial, distúrbios da condução cardíaca e morte súbita. ^2 , 3^ Neste estudo foi apresentada a comparação de características clínicas e eletrofisiológicas de pacientes com flutter atrial portadores da síndrome do PRKAG2 devido à mutação Arg302Gln, e pacientes com flutter atrial sem o fenótipo da síndrome.

Uma das características mais marcantes do eletrocardiograma dos pacientes portadores da mutação do gene *PRKAG2* é a presença de pré-excitação ventricular, mimetizando a síndrome de WPW. ^1^ É descrito que a incidência de fibrilação atrial na síndrome de WPW é maior que na população geral, estimada entre 10 e 23%, na ausência de cardiopatia estrutural. ^19^ Após a ablação por cateter da via acessória, o risco de arritmia atrial é reduzido significativamente. ^20^ Todavia, é rara na literatura a descrição de flutter atrial em portadores de síndrome de WPW. ^10^ Por sua vez, pacientes portadores de miocardiopatia hipertrófica apresentam elevada incidência de fibrilação atrial. ^15^ A síndrome de PRKAG2 apresenta aspectos fenotípicos comuns a estas duas doenças, porém as características clínicas e prognóstico são peculiares. ^18^ Em relação a taquiarritmias atriais, estima-se que 33% dos portadores da síndrome do PRKAG2 sejam acometidos por fibrilação ou flutter atrial. ^3^ Em nossa casuística, a prevalência de flutter atrial nos portadores com síndrome do PRKAG2 foi de 100% a partir de 50 anos de idade. Portanto, os pacientes apresentaram flutter atrial com idade mais precoce e com prevalência muito superior à encontrada na população geral. ^21^

Quanto às comorbidades e manifestações extracardíacas que concorrem para o aumento da prevalência de arritmias atriais, não foi observada diferença estatisticamente significante em relação à presença de hipertensão arterial sistêmica e disfunção renal entre os dois grupos. Do mesmo modo, fatores reconhecidamente relacionados a maior risco de desenvolvimento de flutter atrial, tais como diabetes mellitus, obesidade, apneia do sono e doença arterial coronariana, estiveram presentes apenas no grupo B. É importante reconhecer, no entanto, que pacientes com síndrome do PRKAG2 podem estar suscetíveis a alterações metabólicas a longo prazo. ^22^ Há descrição na literatura de hipertensão arterial sistêmica em jovens com síndrome do PRKAG2, ^3^ assim como envolvimento renal secundário à nefropatia imunomediada, ^23^ sugerindo envolvimento sistêmico mais importante do que previamente relatado.

A mutação Arg302Gln do gene *PRKAG2* encontrada nos pacientes deste estudo é uma das mais comuns relatadas na literatura. ^2^ Porém, a correlação entre genótipo e fenótipo permanece incerta. Foi observada tendência de pacientes com mutação Arg302Gln a apresentarem maior prevalência de pré-excitação ventricular, síncope e implante de marca-passo em relação a pacientes com mutação Asn488Ile, porém com menor prevalência de hipertrofia ventricular esquerda. ^2^ Como caracteristicamente descrito na síndrome do PRKAG2, observamos que a maioria dos pacientes do grupo A apresentaram bloqueio de ramo direito e bradicardia sinusal. Por outro lado, somente 8% dos pacientes do grupo B apresentavam distúrbio de condução. A bradicardia sinusal é tipicamente progressiva e pode levar à incompetência cronotrópica e implante de marca-passo. Análise de estudo experimental sugere que a enzima AMPK determina a adaptação cardíaca fisiológica ao exercício, através da modulação de canais iônicos e liberação de cálcio em células do nó sinusal. ^24^ Em nosso estudo, outro aspecto peculiar foi a precoce evolução do distúrbio de condução elétrico em quatro pacientes do grupo A, com necessidade de implante de marca-passo.

Outro aspecto de interesse é a abordagem do flutter atrial na síndrome do PRKAG2. Durante estudo eletrofisiológico, observamos presença de via acessória fascículo-ventricular nos quatro pacientes do grupo A, sem evidência de taquicardia atrioventricular ou indução de taquicardia ventricular. Sternick et al. não evidenciaram indutibilidade de arritmias ventriculares malignas no estudo eletrofisiológico. ^14^ É provável que este não seja mecanismo importante de morte súbita na síndrome do PRKAG2. Os pacientes com mutação de nosso estudo eram sintomáticos, sendo indicada a estratégia de controle do ritmo através da ablação por cateter do istmo cavo-tricuspídeo, mantendo-os sem recorrência. Portanto, a ablação do flutter atrial parece ser eficaz nos pacientes portadores da síndrome do PRKAG2. Futuros estudos deverão analisar a indicação preventiva de ablação por cateter do istmo cavo-tricuspídeo em pacientes portadores de síndrome do PRKAG2 submetidos a estudo eletrofisiológico diagnóstico.

### Limitações do estudo

Sendo a síndrome do PRKAG2 uma doença rara, podemos considerar como limitações potenciais deste estudo: estudo retrospectivo, envolvendo única mutação Arg302Gln do gene *PRKAG2* , com número limitado de pacientes, podendo comprometer o poder estatístico e capacidade de extrapolação dos dados. O grupo B foi composto de pacientes que apresentaram flutter atrial, sem o fenótipo da síndrome do PRKAG2, mas não foi feita genotipagem nesses pacientes para excluir a presença da mutação. Visto que portadores da mutação apresentam fenótipo bastante característico, com elevada penetrância, a probabilidade de encontrar a mutação em indivíduos sem o fenótipo é pequena, o que não deve comprometer o resultado do estudo.

## Conclusão

Em comparação a pacientes com flutter atrial sem fenótipo da mutação genética, os pacientes com síndrome do PRKAG2 por mutação Arg302Gln apresentaram flutter atrial com idade mais precoce, associado à alta prevalência de distúrbio de condução cardíaco e necessidade de implante de marca-passo. O circuito eletrofisiológico do flutter atrial típico, dependente do istmo cavo-tricuspídeo, foi passível de tratamento através da ablação por cateter.

Assim, propomos que a presença de flutter atrial em indivíduo jovem, sem outras comorbidades, deva alertar para a possibilidade de doença cardíaca geneticamente determinada, como a síndrome do PRKAG2, especialmente na presença de pré-excitação ventricular e hipertrofia ventricular familiar. A confirmação com teste genético e o rastreamento familiar devem fazer parte da estratégia de manejo.
