# Supplementary material for: Flutter Atrial na Síndrome do PRKAG2: Características Clínicas e Eletrofisiológicas
Source: Arq Bras Cardiol. 2022 Sep 6;119(5):681–8. [Article in Portuguese] doi: 10.36660/abc.20210792 (PMC9750222; doi:10.36660/abc.20210792)
Supplement: Translation

# Translation: Atrial Flutter in PRKAG2 Syndrome: Clinical and Electrophysiological Characteristics

**Authors:** Eduardo Faria Soares de Magalhães, Luiz Pereira de Magalhães, Jussara de Oliveira Pinheiro, Alex Teixeira Guabiru, Roque Aras

**Affiliations:** 1 Brazil Universidade Federal da Bahia – Faculdade de Medicina de Bahia, Salvador, BA – Brazil; 2 Brazil Hospital Universitário Professor Edgard Santos, Salvador, BA – Brazil

**Keywords:** Cardiac Arrhythmias, Atrial Flutter, Hypertrophy, Left Ventricular, Cardiomyopathy, Hypertrophic, Atrioventricular Block, Glycogen Storage Disease

## Abstract

**Background:**

PRKAG2 syndrome is a rare autosomal dominant disease, a phenocopy of hypertrophic cardiomyopathy characterized by intracellular glycogen accumulation. Clinical manifestations include ventricular preexcitation, cardiac conduction disorder, ventricular hypertrophy, and atrial arrhythmias.

**Objective:**

To compare the clinical and electrophysiological characteristics observed in patients with atrial flutter, with and without PRKAG2 syndrome.

**Methods:**

An observational study comparing patients with atrial flutter: group A consisted of five patients with PRKAG2 syndrome from a family, and group B consisted of 25 patients without phenotype of PRKAG2 syndrome. The level of significance was 5%.

**Results:**

All patients in group A had ventricular preexcitation and right branch block, and four had pacemakers (80%). Patients in group A were younger (39±5.4 vs 58.6±17.6 years, p=0.021), had greater interventricular septum (median=18 vs 10 mm; p<0.001) and posterior wall thickness (median=14 vs 10 mm; p=0.001). In group A, four patients were submitted to an electrophysiological study, showing a fasciculoventricular pathway, and atrial flutter ablation was performed in tree. All patients in group B were submitted to ablation of atrial flutter, with no evidence of accessory pathway. Group B had a higher prevalence of hypertension, diabetes mellitus, coronary artery disease and sleep apnea, with no statistically significant difference.

**Conclusion:**

Patients with PRKAG2 syndrome presented atrial flutter at an earlier age and had fewer comorbidities when compared to patients with atrial flutter without mutation phenotype. The occurrence of atrial flutter in young individuals, especially in the presence of ventricular preexcitation and familial ventricular hypertrophy, should raise the suspicion of PRKAG2 syndrome.

## Introduction

PRKAG2 syndrome is a rare genetic disease of autosomal dominant inheritance caused by mutations in the gene encoding the γ_2_subunit of AMP-activated protein kinase (AMPK).^1 , 2^ The main histopathological finding in affected patients’ hearts is intracellular myocardial glycogen deposition, which may trigger electrophysiological and structural cardiac changes that mimic Wolff-Parkinson-White syndrome (WPW) and hypertrophic cardiomyopathy.^3^ Studies indicate that the prevalence of PRKAG2 syndrome is 0.23 to 1% of patients with suspected hypertrophic cardiomyopathy.^3 , 4^ The incidence of PRKAG2 syndrome may be underestimated in clinical practice, as many cases are mistakenly diagnosed as sarcomeric hypertrophic cardiomyopathy.

The phenotypic manifestation of PRKAG2 syndrome has great variability, consisting of ventricular preexcitation, left ventricular hypertrophy, conduction system disorders, and atrial tachyarrhythmias.^5^ The early identification of PRKAG2 syndrome is of particular interest, as it is related to a high risk of progression to complete atrioventricular block requiring pacemaker implantation and sudden death.^6^

Concerning atrial tachyarrhythmias, previous studies have shown that atrial flutter is less common than atrial fibrillation in the general population, affecting more men.^7^ From the electrocardiographic point of view, it is typically characterized by the presence of F waves in the lower leads and an approximate atrial frequency of 300 bpm.^8^ The electrophysiological mechanism of atrial flutter involves macroreentry in atria, using slow, anatomical or functional conduction areas. The incidence increases with age, being less than 5 cases per 100,000 inhabitants among people under 50 but reaching almost 600 cases per 100,000 inhabitants among those over 80.^9^ Although WPW syndrome and hypertrophic cardiomyopathy are related to increased prevalence of atrial fibrillation, the association of atrial flutter and ventricular pre-excitation is a rare phenomenon.^10 , 11^ In PRKAG2 syndrome, there are reports in the literature of the appearance of atrial flutter in patients with ventricular pre-excitation,^12^ but there are few data on clinical and electrophysiological behavior of atrial flutter in the disease. Although atrial flutter may be the first manifestation of PRKAG2 syndrome, a delayed diagnosis of the syndrome can occur, especially when there is no evident ventricular hypertrophy.^13^

This study aimed to compare the clinical, electrocardiographic and electrophysiological characteristics observed in patients with atrial flutter, with and without PRKAG2 syndrome. Members of a family with atrial flutter and previously genotyped with the Arg302Gln mutation of the *PRKAG2* gene were analyzed and compared with a control group of patients with atrial flutter without the phenotype of PRKAG2 syndrome.

## Methods

### Study participants

This is an observational, retrospective, comparative study involving patients with atrial flutter, selected by convenience sampling. For this, the following criteria were selected: (1) patients with Arg302Gln mutation of the *PRKAG2* gene who developed atrial flutter; (2) patients with records of typical atrial flutter from our arrhythmia service in the last 5 years. Patients were divided into two groups. Group A was composed of five patients from the same family accompanied in our service, composed of 16 members with PRKAG2 syndrome, previously genotyped,^14^ who developed atrial flutter, with a mean follow-up time of 15.1 ± 2.9 years. Group B included 25 patients with typical atrial flutter, without the phenotype of PRKAG2 syndrome, consecutively submitted to catheter ablation from 2015 to 2020.

Data were obtained from physical examination, laboratory tests, electrocardiogram, echocardiogram, and electrophysiological study. Short PR interval on electrocardiogram was determined when less than 120 ms. The ventricular pre-excitation pattern was defined by the association of short PR interval with increased QRS duration (> 110 ms) or delta wave. The diagnostic criterion of atrial flutter was previously established. Typical atrial flutter on ECG was defined by the presence of negative F waves in IDI, DIII and aVF and positive in V1. The ECG was performed in 12 leads at a speed of 25mm/s, gain of 10mm:1mV and filter from 0.05Hz to 15Hz.^8^

The echocardiogram diagnosis of left ventricular hypertrophy was established against the thickness of the interventricular septum or posterior wall of the left ventricle ≥ 13 mm, with no other apparent cause.^15^ The electrophysiological study and catheter ablation were performed according to previously described techniques, with atrial and ventricular stimulation, using multipolar diagnostic catheters and an 8 mm irrigated or tip ablation catheter. The cavotricuspid isthmus ablation was performed with bidirectional block and demonstration of atrial double potential (> 100 ms).^16^

### Statistical analysis

After collecting the information, the data were stored in an Excel spreadsheet and submitted to statistical analysis, performed with R core Team software (Vienna, Austria) for Windows, free of charge. The descriptive statistical analysis expressed categorical variables in absolute frequency and percentage (%). Continuous variables were expressed by mean ± standard deviation or median (interquartile interval) in cases of non-normal distribution. Data normality was evaluated using the Shapiro-Wilk test. Fisher’s exact test was used to compare categorical variables. As appropriate, continuous variables were compared by student’s t-test for independent samples or Mann-Whitney U test. A value of p< 0.05 was considered significant.

### Ethical issues

The Research Ethics Committee approved the study under number 3.044.277, and a free and informed consent form was obtained from the participants.

## Results

### Clinical characteristics

The clinical, electrocardiographic and echocardiographic characteristics of patients in group A with PRKAG2 syndrome are in Table 1 . The analysis of the family heredogram ( Figure 1 ) shows the pattern of autosomal dominant inheritance of the disease, with a report of three sudden unexplained deaths in the family, in individuals with a median age of 38 years. In all five patients included in this study, atrial flutter was identified as the first clinical manifestation of the disease, with a mean age at diagnosis of 39±5.4 years. On the electrocardiogram, all patients in group A presented an electrocardiographic pattern compatible with ventricular preexcitation, associated with right branch block (BRD) in four (80%). Figure 2A shows a typical electrocardiogram of a patient with *PRKAG2* mutation in sinus rhythm, and Figure 2B demonstrates atrial flutter rhythm tracing. Progression to sinus node dysfunction or atrioventricular block led to pacemaker implantation in 4 (80%), with a mean age at implantation of 44 ± 6 years.

**Table 1:** Clinical characteristics of group A

Patient	Sex	Age	Age at diagnosis	Symptoms	Atrial arrhythmia	PM	LVH
II:5	M	56	30	Palpitation	AFL, AF	+	+
II:6	M	60	42	Syncope, palpitation	AFL, AF	+	+
II:7	F	58	40	Syncope	AFL	+	+
II:10	M	53	44	Presyncope, palpitation	AFL	+	+
III:18	M	43	39	Palpitation	AFL	-	-

*M: male; F: female; PM: pacemaker; AFL: atrial flutter; AF: atrial fibrillation; LVH: left ventricular hypertrophy.*

**Figure 1:** Heredogram of the family with PRKAG2 syndrome, with identification of the five patients with atrial flutter. PM: pacemaker; ICD: cardioverter implantable defibrillator. Individuals tested for a PRKAG2 mutation are identified as carriers (+) or non-carriers (-). A patient underwent ICD implantation due to a misdiagnosis of hypertrophic cardiomyopathy.

**Figure 2:** A) Initial electrocardiogram of patient III:15, evidencing ectopic atrial rhythm and the aspect of ventricular preexcitation, with short PR interval followed by pseudo-delta wave, and QRS complex with right bundle block morphology. B) Electrocardiogram of patient II:10 with the typical pattern of PRKAG2 syndrome, showing atrial flutter with 2:1 conduction.

Group B consisted of 25 patients with typical atrial flutter, 19 (76%) men, six (24%) with a mean age at diagnosis of 58.6±17.6 years, and one (4%) pacemaker-carrying by sinus node dysfunction. Atrial septal defect was documented in three (12%), and atrial flutter-induced tachycardiomyopathy in three (12%). Only two (8%) group B patients had right branch block, and none had ventricular pre-excitation.

### Electrophysiological aspects

Four patients from group A underwent electrophysiological study, three of them male. Short AH and HV intervals were recorded, with fixed HV (median=30 ms), during basic rhythm and rapid atrial stimulation ( Figure 3 ). The Wenckebach point was obtained in four patients, with a mean of 302.5 ± 31 ms. The test with adenosine was performed, with a record of anterograde and retrograde AV block. Decremental ventricle-atrial retrograde conduction was observed during ventricular stimulation in all. The findings were compatible with the presence of fasciculoventricular accessory pathway. Three patients underwent atrial flutter ablation during the procedure, and an arrhythmogenic circuit dependent on the cavotricuspid isthmus was demonstrated. Success was achieved in 100%, without recurrence after 18 months. All patients in Group B were submitted to ablation with a catheter of the typical atrial flutter (cavotricuspid isthmus). The presence of an accessory pathway was not evidenced.

**Figure 3:** Tracing of the electrophysiological study of patient II:10. 5-lead electrocardiogram and basic intervals during atrial stimulation, demonstrating HV = 33 ms. AV: atrium-ventricular; A: atrial electrogram; H: His electrogram; V: ventricular electrogram.

### Comparative aspects of groups A and B

The clinical, electrocardiographic, and echocardiographic characteristics of groups A and B ( Table 2 ) were compared. The mean age at the diagnosis of atrial flutter in group A was significantly lower than in group B (39 ± 5.4 vs. 58.6 ± 17.6 years; p = 0.021). Among the symptoms, a higher prevalence of syncope/presyncope was observed in group A (p = 0.004). Risk factors established for the development of atrial flutter, such as arterial hypertension, diabetes mellitus, sleep apnea, obesity and coronary artery disease, were more prevalent in group B but without statistical significance. There was no statistically significant difference between the groups concerning renal function.

**Table 2:** Comparative result of the two groups’ clinical, electro and echocardiographic characteristics

Characteristics	Group A n = 5	Group B n = 25	p
Age (years)	54 ± 6.7	60 ± 17.2	0.422
Age at diagnosis (years)	39.0 ± 5.4	58.6 ± 17.6	0.021
Male	4 (80)	19 (76)	0.999
Syncope	3 (60)	1 (4)	0.009
Hypertension	3 (60)	16 (64)	0.999
DM	0 (0)	3 (12)	0.999
Sleep apnea	0 (0)	1 (4)	0.999
Obesity, BMI > 30 kg/m^2^	0 (0)	8 (32)	0.287
CAD	0 (0)	4 (16)	0.999
Creatinine clearance (mL/min/1,73m^2^)	77.2 (60.7-81.5)	84.4 (66.0-102.8)	0.275
LA (mm)	42 (38-47)	40 (38-42)	0.435
SW (mm)	18 (14-26)	10 (9-11)	<0.001
PW (mm)	14 (11-15)	10 (9-11)	0.001
LVDD (mm)	46 (44-50)	50 (47-55)	0.124
EF (%)	71 (60-76)	66 (59-69)	0.223
LVH	4 (80)	1 (4)	0.009
HR (bpm)	52 (44-57)	62 (56-75)	0.007
PR interval (ms)	100 (100-110)	160 (140-188)	<0.001
QRS (ms)	120 (100-140)	90 (90-93)	0.001
RBB	3 (60)	2 (8)	0.022
CAVB	3 (60)	0 (0)	0.002
Pacemaker	4 (80)	1 (4)	0.001

*Continuous variables were expressed as mean ±, or median (interquartile interval). Categorical variables were expressed as n (%). DM: diabetes mellitus; CAD: coronary artery disease; BMI: body mass index; HR: heart rate; RBB: right branch block; CAVB: complete atrioventricular block; LA: left atrium; SW: septal wall; PW: posterior wall; LVDD: left ventricular diastolic diameter; EF: ejection fraction; LVH: left ventricular hypertrophy.

On baseline electrocardiogram in sinus rhythm, it was observed that group A had lower heart rate, shorter PR interval and higher prevalence of BRD. Regarding echocardiographic characteristics, left ventricular hypertrophy was observed in 80% of patients in group A and only 6% of group B (p=0.001). There was no statistically significant difference in relation to the ejection fraction and left atrium size.

In addition, total atrioventricular block was observed only in patients in group A (80% vs 0%, p< 0.001), as well as more often required PM implantation in relation to group B (80% vs 8%, p = 0.002).

## Discussion

PRKAG2 syndrome is a rare phenocopy of hypertrophic cardiomyopathy, mimicking WPW syndrome.^17^ However, the diagnostic distinction is crucial since the natural history, prognosis and treatment strategies are markedly different.^18^ Clinical manifestations of PRKGA2 syndrome include atrial tachyarrhythmias such as atrial flutter, conduction system disorders, and sudden death.^2 , 3^ This study compared the clinical and electrophysiological characteristics of patients with atrial flutter with PRKAG2 syndrome due to the Arg302Gln mutation and patients with atrial flutter without the syndrome phenotype.

One of the most striking characteristics of the electrocardiogram of patients with the *PRKAG2* gene mutation is the presence of ventricular pre-excitation, mimetizing WPW syndrome.^1^ It is described that the incidence of atrial fibrillation in WPW syndrome is higher than in the general population, estimated between 10 and 23%, in the absence of structural heart disease.^19^ After catheter ablation of the accessory pathway, the risk of atrial arrhythmia is significantly reduced.^20^ However, descriptions of atrial flutter in patients with WPW syndrome are rare in the literature.^10^ On the other hand, patients with hypertrophic cardiomyopathy have a high incidence of atrial fibrillation.^15^ PRKAG2 syndrome presents phenotypic aspects common to these two diseases, but the clinical characteristics and prognosis are peculiar.^18^ Regarding atrial tachyarrhythmias, it is estimated that 33% of *PRKAG2* mutation carriers are affected by atrial fibrillation or atrial flutter.^3^ In our series, the prevalence of atrial flutter in patients with PRKAG2 syndrome was 100% from 50 years of age. Therefore, patients presented atrial flutter at an earlier age, with a much higher prevalence than in the general population.^21^

As for comorbidities and extracardiac manifestations that contribute to the increased prevalence of atrial arrhythmias, no statistically significant difference was observed in relation to the presence of hypertension and renal dysfunction between the two groups. However, it is important to note that patients with PRKAG2 syndrome may be susceptible to metabolic changes in the long term.^22^ There are descriptions in the literature of hypertension in young patients with PRKAG2 Syndrome,^3^ and renal involvement secondary to immunomediated nephropathy,^23^ suggesting that systemic impairment is far more important than previously described.

The Arg302Gln mutation of the *PRKAG2* gene found in the patients of this study is one of the most commonly reported in the literature.^2^ However, the correlation between genotype and phenotype remains uncertain. Patients with Arg302Gln mutation presented a higher prevalence of ventricular preexcitation, syncope and pacemaker implantation compared to patients with Asn488Ile mutation, but with a lower prevalence of left ventricular hypertrophy.^2^ As characteristically described in PRKAG2 syndrome, we observed that most patients in group A presented right branch block and sinus bradycardia. On the other hand, only 8% of patients in group B had conduction disorder. The sinus bradycardia in *PRKAG2* mutation is typically progressive and can lead to chronotropic incompetence and the need for pacemaker implantation. Analysis from an experimental study suggests that AMPK determines cardiac physiological adaptation to exercise through modulation of ion channels and calcium release in sinoatrial cells.^24^ In our study, another peculiar aspect was the early evolution of the electrical conduction disorder in four patients in group A, requiring pacemaker implantation.

Another aspect of interest is the approach to atrial flutter in PRKAG2 syndrome. During the electrophysiological study, we observed the presence of a fasciculoventricular accessory pathway in the four patients of group A, without evidence of atrioventricular tachycardia or induction of ventricular tachycardia. Sternick et al. showed no inducibility of malignant ventricular arrhythmias in the electrophysiological study,^14^ suggesting that this is not an important mechanism of sudden death in PRKAG2 syndrome. Patients with *PRKAG2* mutation in our study were symptomatic, and the chosen strategy was rhythm control through catheter ablation of the cavotricuspid isthmus, which prevented further recurrence. Therefore, atrial flutter ablation seems effective in patients with PRKAG2 syndrome. Future studies should analyze a possible role for prophylactic cavotricuspid isthmus ablation in patients with PRKAG2 syndrome undergoing diagnostic electrophysiological study.

### Study limitations

Since PRKAG2 syndrome is a rare disease, we consider as potential limitations of this study: the retrospective analysis involving only the Arg302Gln mutation of the *PRKAG2* gene and a limited number of patients, which may compromise the statistical power and extrapolation capacity of the data. Group B was composed of patients who presented atrial flutter without the phenotype of PRKAG2 syndrome, but genotyping was not performed in these patients to exclude the presence of the mutation. Since *PRKAG2* mutation carriers have a very distinct phenotype with high penetrance, the probability of finding the mutation in individuals without PRKAG2 syndrome phenotype is low; thus, it does not represent a significant limitation for the study.

## Conclusion

Compared to patients with atrial flutter without a genetic mutation phenotype, patients with PRKAG2 syndrome caused by Arg302Gln mutation presented atrial flutter at an earlier age, associated with a high prevalence of cardiac conduction disorder and need for pacemaker implantation. The typical atrial flutter electrophysiological circuit, dependent on the cavotricuspid isthmus, was amenable to treatment through catheter ablation.

Thus, we propose that the presence of atrial flutter in a young individual without other comorbidities should alert to the possibility of genetically determined heart disease, such as PRKAG2 syndrome, especially in the presence of ventricular pre-excitation and familial left ventricular hypertrophy. Confirmation with genetic testing and family screening should be part of the management strategy.
